# Update on Existing Care Models for Chronic Kidney Disease in Low- and Middle-Income Countries: A Systematic Review

**DOI:** 10.1177/20543581221077505

**Published:** 2022-03-02

**Authors:** Victoria Nkunu, Natasha Wiebe, Aminu Bello, Sandra Campbell, Elliot Tannor, Cherian Varghese, John Stanifer, Marcello Tonelli

**Affiliations:** 1 Faculty of Medicine and Dentistry, University of Alberta, Edmonton, AB, Canada; 2 John W. Scott Health Sciences Library, University of Alberta, Edmonton, Canada; 3 Department of Medicine, Komfo Anokye Teaching Hospital, Kumasi, Ghana; 4 Department of Noncommunicable Diseases, World Health Organization, Geneva, Switzerland; 5 Department of Medicine, Duke University, Durham, NC, USA; 6 Cumming School of Medicine, University of Calgary, AB, Canada

**Keywords:** chronic kidney disease, developing countries, patient care, global health, LMICs

## Abstract

**Background:**

Approximately 78% of chronic kidney disease (CKD) cases reside in low- and middle-income countries (LMICs). However, little is known about the care models for CKD in LMICs.

**Objective:**

Our objective was to update a prior systematic review on CKD care models in LMICs and summarize information on multidisciplinary care and management of CKD complications.

**Design:**

We searched MEDLINE, EMBASE, and Global Health databases in September 2020, for papers published between January 1, 2017, and September 14, 2020. We used a combination of search terms, which were different iterations of CKD, care models, and LMICs. The World Bank definition (2019) was used to identify LMICs.

**Setting:**

Our review included studies published in LMICs across 4 continents: Africa, Asia, North America (Mexico), and Europe (Ukraine). The study settings included tertiary hospitals (n = 6), multidisciplinary clinics (n = 1), primary health centers (n = 2), referral centers (n = 2), district hospitals (n = 1), teaching hospitals (n = 1), regional hospital (n = 1), and an urban medical center (n = 1).

**Patients:**

Eighteen studies met inclusion criteria, and encompassed 4679 patients, of which 4665 were adults. Only 9 studies reported mean eGFR which ranged from 7 to 45.90 ml/min/1.73 m^2^.

**Measurements:**

We retrieved the following details about CKD care: funding, urban or rural location, types of health care staff, and type of care provided, as defined by Kidney Disease Improving Global Outcomes (KDIGO) guidelines for CKD care.

**Methods:**

We included studies which met the following criteria: (1) population was largely adults, defined as age 18 years and older; (2) most of the study population had CKD, and not end-stage kidney disease (ESKD); (3) population resided in an LMIC as defined by the World Bank; (4) manuscript described in some detail a clinical care model for CKD; (5) manuscript was in either English or French. Animal studies, case reports, comments, and editorials were excluded.

**Results:**

Eighteen studies (24 care models with 4665 patients) met inclusion criteria. Out of 24 care models, 20 involved interdisciplinary health care teams. Twenty models incorporated international guidelines for CKD management. However, conservative kidney management (management of kidney failure without dialysis or renal transplant) was in a minority of models (11 of 24). Although there were similarities between all the clinical care models, there was variation in services provided and in funding arrangement; the latter ranged from comprehensive government funding (eg, Sri Lanka, Thailand), to out-of-pocket payments (eg, Benin, Togo).

**Limitations:**

These include (1) lack of detail on CKD care in many of the studies, (2) small number of included studies, (3) using a different definition of care model from the original Stanifer et al paper, and (4) using the KDIGO Guidelines as the standard for defining a CKD care model.

**Conclusions:**

Most of the CKD models of care include the key elements of CKD care. However, access to such care depends on the funding mechanism available. In addition, few models included conservative kidney management, which should be a priority for future investment.

**Trial registration:**

Not applicable.

## Abrégé

**Contexte:**

Environ 78 % des patients atteints d’insuffisance rénale chronique (IRC) habitent un pays à revenu faible ou intermédiaire (PRFI). On en sait toutefois peu sur les modèles de prise en charge pour l’IRC dans les PRFI.

**Objectifs:**

Nous souhaitions faire la mise à jour d’une revue systématique antérieure qui portait sur les modèles de prise en charge pour l’IRC dans les PRFI. Nous voulions également synthétiser l’information concernant les soins multidisciplinaires en IRC et la prise en charge des complications.

**Conception de l’étude:**

En septembre 2020, les bases de données MEDLINE, EMBASE et Global Health ont été consultées à la recherche d’articles publiés entre le 1er janvier 2017 et le 14 septembre 2020. Nous avons utilisé une combinaison de termes de recherche, incluant différentes itérations d’IRC, de modèles de prise en charge et de PRFI. La définition de la Banque mondiale (2019) a été utilisée pour identifier les PRFI.

**Cadre:**

Nous avons inclus des études publiées dans des PRFI de quatre continents : Afrique, Asie, Amérique du Nord (Mexique) et Europe (Ukraine). Les études avaient été réalisées dans des hôpitaux tertiaires (N = 6), une clinique multidisciplinaire, des centres de soins primaires (N = 2), des centres d’aiguillage (N = 2), un hôpital communautaire, un hôpital universitaire, un hôpital régional et un centre médical urbain.

**Sujets:**

Les 18 études répondant aux critères d’inclusion portaient sur un total de 4 679 patients, dont 4 665 adultes. Neuf études seulement rapportaient un DFGe moyen, lequel s’étendait de 7 à 45,90 ml/min/1,73 m^2^.

**Mesures:**

Les informations suivantes sur les soins en IRC ont été extraites : financement, établissement urbain ou rural, catégories de personnel soignant et type de soins fournis, définis par les recommandations de KDIGO (Kidney Disease Improving Global Outcomes) pour la prise en charge d’IRC.

**Méthodologie:**

Nous avons inclus les études qui répondaient aux critères suivants : (1) la population étudiée était principalement constituée d’adultes (18 ans et plus); (2) la majorité de la population étudiée était atteinte d’IRC et non d’insuffisance rénale terminale (IRT); (3) la population étudiée habitait un PRFI selon la définition de la Banque mondiale; (4) le manuscrit décrivait avec suffisamment de détails un modèle de soins cliniques pour l’IRC; (5) le manuscrit était rédigé en anglais ou en français. Les études sur les animaux, les rapports de cas, les commentaires et les éditoriaux ont été exclus.

**Résultats:**

18 études (24 modèles de soins, 4 665 patients) répondaient aux critères d’inclusion. Sur 24 modèles de soins, 20 avaient impliqué des équipes de soins interdisciplinaires. Les recommandations internationales pour la prise en charge de l’IRC avaient été intégrées à 20 modèles de soins. La prise en charge conservatrice de l’IRC (sans dialyse ni greffe rénale) n’était cependant rapportée que dans une minorité de modèles (11/24). Bien que nous ayons noté des similitudes entre tous les modèles de soins cliniques, des variations ont été observées dans les services fournis et dans les modalités de financement; ces dernières allant du financement public complet (p. ex. : Sri Lanka, Thaïlande) aux versements directs par les patients (p. ex. : Bénin, Togo).

**Limites:**

Les limites comprennent notamment: (1) le manque de détails sur les soins en IRC dans plusieurs études; (2) le faible nombre d’études incluses; (3) l’utilisation d’un modèle de soins dont la définition différait de l’originale présentée par Stanifer et coll.; et (4) l’utilisation des recommandations de KDIGO comme norme pour définir un modèle de soins pour l’IRC.

**Conclusion:**

La plupart des modèles de soins intégraient les éléments clés des soins recommandés pour l’IRC. L’accès à ces soins dépendait toutefois du mécanisme de financement en place. Cependant, peu de modèles intégraient la prise en charge conservatrice de l’IRC, laquelle devrait être une priorité pour de futurs investissements.

## Introduction

Many low- and middle-income countries (LMICs) face a double burden of communicable and non-communicable diseases (NCDs). The estimated global prevalence of chronic kidney disease (CKD) is 13.4%,^1^ and most CKD patients worldwide reside in LMICs. Despite this high and growing burden, LMICs often have limited capacity to manage CKD; financial resources are often limited, other public health issues like vaccination, child and maternal health, environmental sanitation, clean water supply, peace, and security may be prioritized over CKD, and health professionals may lack skills in CKD management.^2^ In a 2018 systematic review, Stanifer et al^3^ summarized the existing literature on CKD models of care in LMICs.^3^ They found that although existing literature demonstrated the effectiveness of national programs to screen for CKD and bolster primary care management of CKD, there was limited data on local programs and their effectiveness in CKD management.^3^ Since the Stanifer review was completed, there has been increasing interest in how CKD is managed in LMICs, and especially in how key elements of CKD management as defined by Kidney Disease Improving Global Outcomes (KDIGO) guidelines are included (or not) in the most common models of care.

Given the well-known differences in health care delivery between high-income countries and LMICs (as well as among individual LMICs), closing this knowledge gap is prerequisite to implementing effective kidney care programs in LMICs. Closing this gap will allow insights about how existing models can be strengthened and possibly scaled to a national level.

We did a systematic review of published literature to summarize what is known about models of CKD care in LMICs, with particular focus on technical details of local CKD programs like financing, staffing, regulation, and patient selection. Our ultimate goal is to contribute to the limited body of literature on CKD care in LMICs, by highlighting successes and limitations in existing models of care, to inform future efforts to develop CKD care models in LMICs. Our systematic review was similar to Stanifer et al^3^ in terms of its focus on LMICs and inclusion of CKD models of care at the local level. However, Stanifer et al^3^ included national initiatives for identification of CKD, which we did not. In addition, we used the KDIGO guidelines as our framework for defining CKD care, which Stanifer et al^3^ did not. Finally, our systematic review is more current and includes studies which have been conducted since the publication of the Stanifer et al paper^3^ in 2018.

## Methods

Our primary objective was to characterize models of CKD management in LMICs. Using the systematic review of Stanifer and others^3^ as our framework, we specifically sought papers that described, either in full or partial detail, how key services of CKD care (eg, anemia management and mineral and bone disorders) are managed in health facilities in LMICs.

### Study Setting

Our systematic review was focused on LMICs, where a majority of the worldwide CKD cases reside. As CKD prevalence increases, it is anticipated that LMICs will face an increased burden of CKD, so we focused our systematic review on LMICs to evaluate the strengths and weaknesses of existing models of care.

### Concepts and Definitions

We defined a CKD model of care as any existing system of care that was used clinically to manage CKD, as defined by the KDIGO guidelines for CKD management.^4^

This definition is different from the definition used by Stanifer et al^3^ because we sought to use a more inclusive definition of “model of care” based on KDIGO guidelines, which focuses on models of care that manage CKD in a multidisciplinary care setting, thus allowing for observing and measuring clinical outcomes with the goal of improving overall care.

### Search Strategy

Our expert librarian (S.C.) conducted a literature search of MEDLINE, EMBASE, and Global Health databases in September 2020, for studies published between January 1, 2017, and September 14, 2020, describing CKD care in LMICs. Search yield from the current review was added to the studies identified by the Stanifer review (January 2000-October 31, 2017). Thus, the pooled search yield included studies from January 1, 2000, to September 14, 2020.

We used combinations of the following search terms and their synonyms, in addition to low- and middle-income search filters, as based on the World Bank LMIC list,^5^ to search the databases for relevant articles: *community health services, health services, primary health care, rural health services, telemedicine, disease management, health promotion, nutrition therapy, community health workers, management, education, multidisciplinary, integrated models, services, renal insufficiency*, and *CKD*.

Animal studies, case reports, comments, and editorials were excluded, and the search was limited to January 2017 to September 14, 2020. No other limits were applied. Details of the database-specific search strategies are included in the Supplementary Material. We did not check the references of secondary research studies/review articles, that is, “snowballing.”

### Study Selection

We included studies which met the following criteria: (1) population was largely adults, defined as age 18 years and older; (2) most of the study population had CKD, and not end-stage kidney disease (ESKD); (3) population resided in an LMIC as defined by the World Bank; (4) manuscript described in some detail a clinical care model for CKD; (5) manuscript was in either English or French.

We excluded papers which were not in English and French, because these were the languages the authors were familiar with. We also excluded studies that did not stratify CKD severity by stage, to ensure that participants with ESKD were not over-represented in our data.

Two reviewers (M.T. and V.N.) independently screened all titles and abstracts generated from the database search for relevant studies. Then 2 reviewers (V.N. and N.W.) randomly selected 10 studies to calibrate the study relevance form (see Supplementary Material), and to make any final changes to the inclusion criteria. The full manuscripts of all included records were reviewed independently and in duplicate (V.N. and N.W.) using the relevance form, and studies that met the inclusion criteria were used for the systematic review. Any disagreements were resolved following discussion between both reviewers, with MT as adjudicator. We used the kappa statistic to estimate inter-rater agreement between both reviewers. Kappa was 0.55.

We also screened the references in the Stanifer et al^3^ systematic review, using the same inclusion criteria.

### Data Extraction and Analysis

V.N. retrieved and recorded details about the study (author, year, country, study objective, population and mean estimated glomerular filtration rate [eGFR], clinical setting, study design, and author findings) and the CKD care program in a pre-formed data extraction table: funding, urban or rural location, types of health care staff, and services (eg, anemia management, mineral and bone disorder management, nutrition and diet counseling, hypertension management, arranging vascular access, discussing modality selection, transplant workup, medication review, financial advice and support, diabetes management, cardiovascular disease care, vaccinations, and conservative kidney management). V.N. contacted study authors by email, Skype, Zoom, or phone call for any missing information.

Given the heterogeneity in the methods and design of the studies, we could not perform a meta-analysis with the data or risk of bias assessment of the studies. Instead, we extracted details about CKD management from the studies, and presented them in a narrative format, as a snapshot of CKD care in LMICs today.

This systematic review was conducted and reported according to MOOSE guidelines.^6^

## Results

### Study Selection

Our search of EMBASE, Global Health, and MEDLINE yielded 567, 28, and 101 records, respectively. After removing duplicates, 611 records were identified for screening. We then added 6 records from the Stanifer et al^3^ systematic review which met our inclusion criteria, resulting in a total of 617 records.

**Figure 1.**
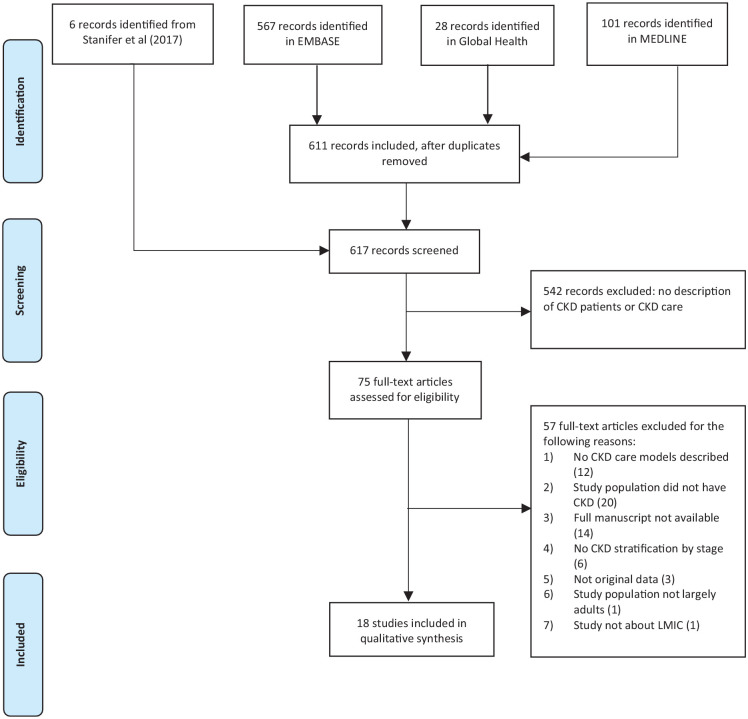
PRISMA diagram.

Most records (N = 542) were excluded because they did not provide a description of a CKD care model. We reviewed the full text of the remaining 75 records and excluded 57 for the following reasons: (1) no CKD care model was described (n = 12), (2) the study population did not have CKD (n = 20), (3) the full manuscript was not available (n = 14), (4) there was no CKD stratification by stage (n = 6), (5) the article did not have original data (n = 3), (6) the study population was not largely adults (n = 1), and (7) the model of care was not in an LMIC (n = 1). The remaining 18 records (comprising 18 distinct studies and 24 CKD care models) were included in the systematic review (Figure 1). Even though we set limitations on the language of the studies to English and French in our inclusion criteria, in the end, we did not exclude any studies based on language.

### Study Characteristics

Details of study characteristics are in outlined in Table 1. A total of 4679 CKD patients were identified, with most (n = 4665) as adults. Studies were published between 2003 and 2020, with a majority between 2017 and 2019. Studies were conducted in Africa, Asia, North America (Mexico), and Europe (Ukraine).

**Table 1.** Study Characteristics.

Author, country	Objective	Participants (mean eGFR in ml/min/1.73 m^2^)	Setting	Study design	Findings
Senanayake et al^7^ Sri Lanka	To assess how much CKD patients pay out-of-pocket to access health care service in the Anuradhapura district	1174 patients with CKD, including patients with ESKD on dialysis (31.8)	19 Medical Officer of Health areas in the district	Community-based cross-sectional study: Questionnaires about out-of-pocket health expenses	Patients with CKD spend a significant portion of their income accessing health care services in the Anuradhapura district
Ahlawat et al^8^ India	To evaluate the cost of treating CKD in an outpatient department at a tertiary hospital	150 patients with CKD (males 17.3, females 19.5)	Government-owned tertiary hospital	Cross-sectional study: review of clinical records and in-person interviews	Patients with CKD had high out-of-pocket expenses for health care. The direct cost of CKD care was influenced by hemodialysis, financial support from employer, smoking, co-morbidities, and whether or not the patient had ESKD
Tannor et al^9^ Ghana	To determine the quality of life of patients with moderate to severe CKD, and its predictors	202 adult patients with moderate to severe CKD, not on dialysis (median eGFR: 7)	Kidney outpatient clinic at a tertiary hospital	Cross-sectional study: In-person interviews	Moderate to severe CKD patients have poor quality of life. Anemia and low-socioeconomic status appear to be closely linked to poor quality of life
Garcia-Garcia et al^10^ Mexico	To report CKD outcomes from a nurse-led CKD clinic in Guadalajara, Mexico between 2008 and 2011	353 patients with CKD (31.7)	Multidisciplinary outpatient CKD clinic	Cross-sectional study: Review of clinical records	Nurse-led multidisciplinary clinic improved clinical outcomes of CKD patients, ie, medication compliance, blood pressure, serum glucose, serum cholesterol, anemia, serum calcium, and phosphate
Cueto-Manzano et al^11^ Mexico	To compare the efficacy of a multiple intervention model vs a conventional health care model in modifying negative lifestyle habits (ie, modifiable CKD risk factors)	300 patients with diabetes and stages 1-2 CKD; results from 39 reported (NR)	Primary Health Care (Family Medicine Units)	Prospective cohort study: Educational interventions against modifiable risk factors in CKD	The multi-intervention model compared with the conventional health care model resulted in better control of lifestyle factors (dietary habits, exercise, emotional management), knowledge of CKD and adherence to treatment in patients with type 2 diabetes who have CKD stage 1-2
Jiamjariyapon et al^12^ Thailand	To evaluate the impact of an Integrated Kidney Care model in slowing down the progression of CKD	441 patients with CKD stages 3-4 (41)	Multidisciplinary teams at district hospitals	Community-based cluster randomized controlled trial	Integrated CKD care, involving multidisciplinary teams resulted in decreased progression of CKD
Tungsanga et al^13^ Thailand	To describe a multidisciplinary model of kidney care in a tertiary hospital	17 patients with CKD (mean range, 24.4-25)	Multidisciplinary outpatient clinic at a tertiary hospital	Prospective cohort study: Periodic monitoring of serum biochemistries, creatinine clearance and 24-hour urine protein over a 4-year period	A multidisciplinary approach, with a focus on nutrition counseling and medical therapy is effective in slowing CKD progression
Yang et al^14^ Multiple countries	To characterize different models of CKD and ESKD care around the world	Not applicable (NR)	15 countries—a mix of high-income, middle-income, and low-income countries	Narrative review—Case studies from 15 countries on CKD and ESKD care	Countries are addressing the rising prevalence of CKD and ESKD in innovative ways, which incorporate available human and financial resources
Gapira Bimenyimana et al^15^ Rwanda	To ascertain the level of CKD knowledge and perceptions of inpatient CKD management among nurses at 2 public referral hospitals	120 nurses working in the emergency department, internal medicine ward, and dialysis unit (not applicable)	2 public referral hospitals in the capital city, Kigali	Non-experimental descriptive correlational study: Self-administered questionnaire to nurses in 3 hospital departments	There is a moderate level of knowledge regarding CKD care among nurses at the 2 referral hospitals. Nurses working in the kidney/dialysis unit had a better understanding of CKD inpatient management compared with nurses in the emergency department and on the internal medicine unit.
Jafar ^16^India	To investigate the experiences of patients and health care workers in accessing CKD care. Also, to identify barriers and facilitators that influence access to CKD care.	21 stakeholders (14 health care providers, 5 patients, and 2 district-level officials) (NR)	4 primary health centers serving 2 villages each	Cross-sectional study: One-on-one interviews and focus group discussions	Although the stakeholders were generally aware of the rising prevalence of CKD, this did not translate to increased referral for CKD care, due to multiple systemic barriers, such as poor CKD awareness among patients, financial burden of CKD, and inadequate mechanisms for referral and follow-up of CKD.
Kabinga ^17^Kenya	To assess the care experienced by patients prior to initiating dialysis at a tertiary hospital	82 patients on maintenance hemodialysis for at least 3 months (NR)	Teaching hospital	Cross-sectional study: Interviews and questionnaires	Hypertension and diabetes were major precursors to ESKD in most patients. However, follow-up in hypertension and diabetes clinics did not result in better CKD care. Multidisciplinary care is needed for effective CKD management.
Kuryata et al^18^ Ukraine	To study blood pressure control among patients with non-dialysis-dependent CKD referred to a CKD clinic.	365 patients with CKD stages 1-3, followed in primary care but requiring re-examination/revision of treatment plan (median 79.3)	Regional hospital	Retrospective non-interventional cross-sectional study: BP measurement and monitoring of patients with CKD	Hypertension is highly prevalent among patients with CKD in ambulatory care.
Kankarn ^19^Thailand	To determine the efficacy of self-management and case management vs traditional CKD care	95 adults with CKD stages II-IV in the intervention group and 97 adult patients with CKD stages II-IV in the control group (45.90)	5 urban medical centers in Northeastern Thailand	Community-based cluster randomized control study	Self-management and case management results in slower progression of CKD, when compared with traditional CKD care.
Zhang ^20^China	A description of how a kidney management clinic was established	1000 patients with CKD (NR)	Kidney management clinic in a tertiary center	Prospective cohort study	The medical care model which was employed by the kidney clinic, where nephrologists play a major role in patient care, needs to be revised to include a larger role for multidisciplinary health staff, like nurses and dieticians
Fogazzi et al^21^ Togo	To describe a voluntary-based kidney care program in south Togo and north Benin	NR (NR)	Hospital belonging to the religious Order of Saint John in rural Togo	Cross-sectional study: questionnaires and chart reviews	Patients presented with variety of kidney conditions, which were difficult to manage appropriately due to financial constraints and lack of diagnostic and therapeutic resources
Fogazzi et al^21^ Benin	To describe a voluntary-based kidney care program in south Togo and north Benin	147 adults and 14 children with kidney impairment (NR)	Hospital belonging to the religious Order of Saint John in rural Benin	Cross-sectional study: questionnaires and chart reviews	Patients presented with variety of kidney conditions, which were difficult to manage appropriately due to financial constraints and lack of diagnostic and therapeutic resources
Njeri ^22^Kenya	To evaluate the frequency and predictive factors for MRPs in a tertiary hospital	60 adult patients with CKD (NR)	Tertiary hospital	Cross-sectional study: Chart reviews and structured interviews	There was a high frequency of MRPs in patients with CKD at tertiary hospitals. Multiple factors were associated with MRPs, including the patient’s CKD stage, co-morbidities, and polypharmacy
Bello et al^23^ Nigeria	To describe the mode of presentation to emergency services among CKD patients	158 adult patients with CKD (NR)	2 tertiary hospitals in Southwest Nigeria	Prospective cohort study: Recruitment from emergency departments at 2 tertiary facilities; kidney outcomes followed in hospital	Many CKD patients were first diagnosed when they presented to the emergency department with ESRD complications requiring urgent initiation of dialysis. This was associated with increased mortality.
Marie Patrice ^24^Cameroon	To estimate the prevalence of late referrals to a nephrologist in CKD care and its associated factors	130 adult patients diagnosed with CKD at 2 referral centers (median 13.0)	2 referral hospitals in Douala, Cameroon	Descriptive cross-sectional study—Patient interviews and questionnaires	There is a high prevalence of late presentation to a nephrologist (approximately three-fourths CKD patients). The main physician-related factor was failure to screen for CKD, while the main patient-related factors were failure to follow through with the referral, and failure to seek care in a hospital.

*Note.* eGFR = estimated glomerular filtration rate; CKD = chronic kidney disease; ESKD = end-stage kidney disease; MRP = medication-related problems, NR = not reported.

Of the 18 studies selected for the systematic review, 10 were cross-sectional studies, 4 were prospective cohort studies, 2 were cluster randomized controlled trials, 1 was a narrative review, and 1 was described as non-experimental descriptive correlational study. Nine studies did not report mean eGFR. Of the remaining 9, eGFR ranged from 7 to 45.90 ml/min/1.73 m^2^.

A wide variety of terms were used to describe the locations where the studies were conducted. They were tertiary hospitals (n = 6), multidisciplinary outpatient CKD clinic (n = 1), primary health care centers (n = 2), referral hospitals (n = 2), district hospital (n = 1), teaching hospital (n = 1), regional hospital (n = 1), urban medical center (n = 1), hospitals belonging to a religious order (n = 1) and an administrative district comprising of 19 medical officer of health areas (n = 1). One paper (Yang et al^14^) included studies from multiple countries, without a description of the locations where the individual studies were conducted.

### Characteristics of Kidney Programs in LMICs

**Figure 2.**
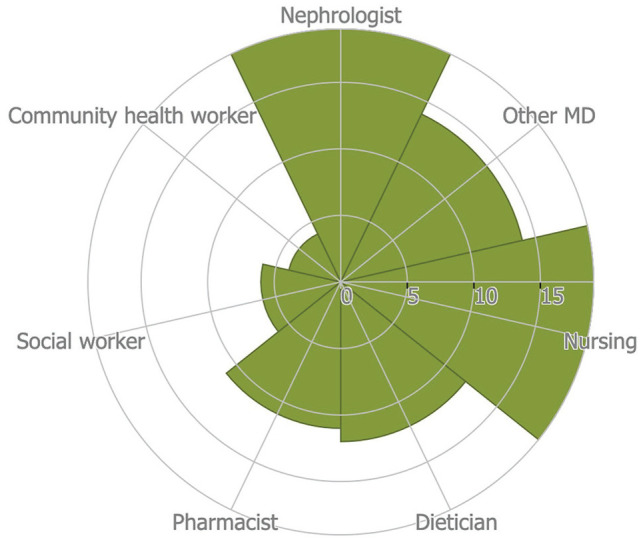
Number and type of staff represented across the CKD Programs. *Note.* A Rose Diagram illustrating the number and types of staff involved in CKD care across the 24 models of care included in the systematic review. CKD = chronic kidney disease.

Thirteen of the CKD programs were based in urban centers, while the remainder (except for one, where location was not indicated) were based in rural areas. The characteristics were examined and reported using categories of workforce, funding, services, and other management support capacity. The study by Yang et al,^14^ which is a narrative review of multiple CKD care models, will be described separately. Details of the characteristics of the kidney programs are outlined in Table 2 and Figure 2.

**Table 2.** Characteristics of the Kidney Programs.

Author	Funding for CKD care	Location	Health care staff							
			Nephrologist	Other MD (family doctors, generalists, trainees, specialists)	Nursing	Dietician	Pharmacist	Social Worker	Community health worker	Other
Senanayake et al^7^	Government funding	Urban	Y	Y	Y	N	Y	N	N	—
Ahlawat et al^8^	Mostly out-of-pocket. Some coverage from government, private and employment insurance	Urban	Y	Y	Y	N	Y	N	N	—
Tannor et al^9^	Mostly out-of-pocket, with some government funding for medications such as antihypertensives, haematinics, and antidiabetics	Urban	Y	Y	Y	Y	Y	N	N	Referral to family medicine unit for conservative kidney management and rehab
Garcia-Garcia et al^10^	Government funding	Urban	Y	N	Y	Y	N	Y	N	Health psychology specialist
Cueto-Manzano et al^11^	Social security system for workers and their families	Urban	N	Y	N	Y	N	Y	N	Physical trainer
Jiamjariyapon et al^12^	Government funding	Rural	N	Y	Y	Y	Y	N	Y	Physical therapist
Tungsanga et al^13^	Government funding	Urban	Y	Y	Y	Y	Y	Y	N	—
Yang et al^14^—*see countries listed below*	—	—	—	—	—	—	—	—	—	—
*Senegal*	Out-of-pocket	Urban	Y	Y	Y	N	N	N	N	—
*Kenya*	Out-of-pocket/private insurance	Urban	Y	Y	Y	N	N	N	N	—
*Malaysia*	Private-public partnership	Urban	Y	Y	Y	Y	Y	Y	N	—
*Malawi*	Government funding	Urban	Y	Y	Y	N	N	N	N	Clinical assistants
*China*	Government funding	Urban	Y	N	Y	Y	Y	N	N	Pathologist
*Ghana*	Out-of-pocket	Urban	Y	Y	Y	Y	Y	N	Y	Clinical psychologist
Gapira Bimenyimana et al^15^	Out-of-pocket/private insurance	Urban	Y	N	Y	Y	Y	Y	N	—
Jafar^16^	Government funding and out-of-pocket	Rural	N	Y	Y	N	N	N	Y	
Kabinga^17^	Out-of-pocket	Urban	Y	Y	Y	N	N	N	N	Counselor—provides psychological care
Kuryata et al^18^	Out-of-pocket	Urban	Y	N	Y	Y	N	N	N	—
Kankarn^19^	Government funding	Urban	N	Y	Y	Y	Y	Y	Y	Physical therapist
Zhang^20^	Government funding	NR	Y	N	Y	Y	N	N	N	—
Fogazzi et al^21^—Togo and Benin	Out-of-pocket	Rural	Y	NR	NR	NR	NR	NR	NR	—
Njeri^22^	Out of pocket/private insurance	NR	Y	NR	Y	NR	Y	NR	NR	—
Bello et al^23^	Out-of-pocket	Urban	Y	NR	NR	NR	NR	NR	NR	—
Marie Patrice^24^	Out-of-pocket	Urban	Y	NR	NR	NR	NR	NR	NR	

*Note.* CKD = chronic kidney disease; eGFR = estimated glomerular filtration rate; MD = medical doctor; Y = yes (green); N = no (red); NR = not reported (grey).

Nephrologists were an intrinsic part of the model of CKD care in 13 out of 17 studies describing CKD care models (excluding Yang et al^14^). For the other 4 studies, referrals to nephrologists at other facilities were made if needed. Chronic kidney disease programs described in 9 studies also involved other non-nephrology physicians, including general practitioners, family doctors, and medical trainees. Other health professionals also participated—nurses were reported in 13 studies, dieticians in 9, and pharmacists in 8. Only a minority of studies reported that a social worker and/or a community health worker was affiliated with the CKD program (n = 5 for social workers and n = 3 for community health workers). Physical therapists were included in 3 programs. One program identified a clinical psychologist, while another had a health psychology specialist to provide mental health support.

In the Yang et al^14^ narrative review which explored CKD care in multiple countries, we identified the LMICs in the paper and included them in our analysis. Of the 6 LMICs identified in the Yang paper, all of them had nephrologists and nurses involved in CKD care. Five of them had other non-specialist physicians involved, 3 programs had dieticians, 3 had pharmacists, 1 had a social worker, and 1 had a community health worker.

The funding mechanisms for CKD care varied substantially across countries. Chronic kidney disease care was fully government funded in Sri Lanka,^7^ Mexico,^10,11^ Thailand,^12^ Malawi,^14^ and China.^14^ The other CKD care models had either partial government funding or were completely funded by out-of-pocket payments. In countries like Ghana,^9^ patients typically pay for most of their care out-of-pocket, with the government paying for some medications like antihypertensives and diabetes medications, through the National Health Insurance Scheme (NHIS).^9^ In India and Malaysia, patients pay for CKD care through a combination of funding from the government and private insurance.^8,14^ In countries like Benin,^21^ Togo,^21^ Nigeria,^23^ Senegal,^14^ Kenya,^14^ Rwanda,^15^ Ukraine,^18^ and Cameroon,^24^ CKD care is solely funded through out-of-pocket payments or through insurance purchased by the patient.

### Services Provided in the CKD Clinics in LMICs

**Figure 3.**
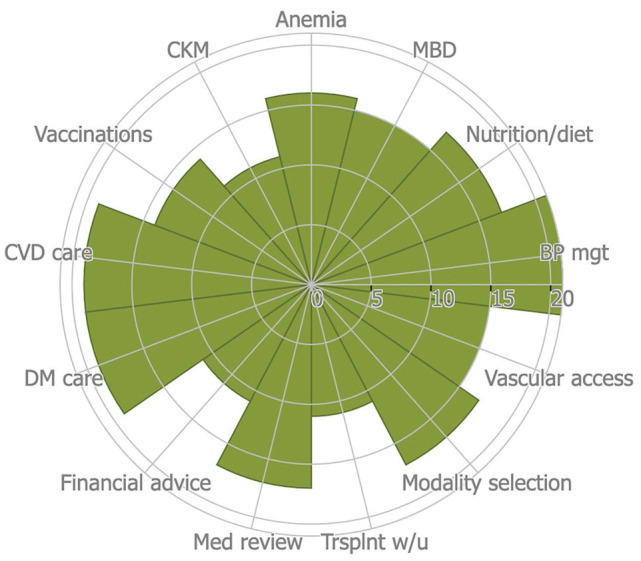
Components of CKD care as defined by KDIGO, provided across the CKD programs. *Note.* A Rose Diagram representing the key elements of CKD care as defined by KDIGO and how often they were provided across the 24 care models included in the systematic review. CKD = chronic kidney disease; KDIGO = Kidney Disease Improving Global Outcomes; CKM = Conservative Kidney Management; MBD = mineral and bone disease; CVD = cardiovascular disease; DM = diabetes mellitus; Med = medication; Trsplnt w/u = transplant workup; BP mgt = blood pressure measurement.

Details of the services provided at the 24 CKD programs are outlined in Table 3 and in Figure 3. The most commonly provided services were those associated with early CKD care such as blood pressure management (n = 21), cardiovascular disease management (n = 19), and diabetes (n = 19). Other services included care of patients with advanced stages of CKD such as education on dialysis modality selection, that is, peritoneal versus hemodialysis (n = 17); vascular access planning, that is, temporary dialysis lines, arteriovenous graft, and fistulas (n = 15); nutrition and dietary counseling, either by physicians or certified dieticians (n = 17); and medication reconciliation (n = 17).

**Table 3.** Services Provided in the CKD Clinics.

Author	Anemia	MBD	Nutrition/diet	BP management	Vascular access	Modality selection	Transplant workup	Medication review	Financial advice or support	DM care	CVD care	Vaccinations	Conservative kidney management	Other
Senanayake et al^7^	Y	Y	Y	Y	Y	Y	Y	Y	Y	Y	Y	Y	Y	Screening programs
Ahlawat et al^8^	Y	Y	Y	Y	Y	Y	N	Y	Y	Y	Y	Y	N	—
Tannor et al^9^	Y	Y	Y	Y	Y	Y	Y	Y	Y	Y	Y	Y (not routine)	Y	Public education
Garcia-Garcia et al^10^	Y	Y	Y	Y	Y	Y	N	Y	Y	Y	Y	N	Y	—
Cueto-Manzano et al^11^	N	N	N	Y	N	N	N	N	N	Y	Y	Y	N	—
Jiamjariyapon et al^12^	N	N	Y	Y	N	N	N	Y	N	Y	Y	N	N	Exercise
Tungsanga et al^13^	Y	Y	Y	Y	Y	Y	Y	Y	Y	Y	Y	Y	Y	Smoking cessation
Yang et al^14^—*see countries listed below*	—	—	—	—	—	—	—	—	—	—	—	—	—	—
*Senegal*	Y	Y	Y	Y	Y	Y	Y	Y	Y	Y	Y	Y	Y	—
*Kenya*	Y	Y	Y	Y	N	N	Y	Y	N	Y	Y	Y	Y	—
*Malaysia*	Y	Y	Y	Y	Y	Y	Y	Y	Y	Y	Y	Y	N	—
*Malawi*	Y	N	Y	Y	Y	Y	Y	Y	N	Y	Y	Y	Y	—
*China*	Y	Y	Y	Y	Y	Y	N	Y	N	Y	Y	N	Y	kidney biopsy, kidney pathology, kidney ultrastructure, kidney ultrasound examination, lab tests
*Ghana*	Y	Y	Y	Y	Y	Y	Y	Y	Y	Y	Y	Y	Y	—
Gapira Bimenyimana et al^15^	Y	Y	Y	Y	Y	Y	Y	Y	Y	Y	Y	Y	Y	—
Jafar^16^	N	N	N	Y	N	N	N	N	N	Y	Y	N	N	
Kabinga^17^	Y	Y	Y	Y	Y	Y	Y	Y	N	Y	Y	Y	N	—
Kuryata et al^18^	Y	Y	Y	Y	Y	Y	Y	Y	Y	Y	Y	Y	N	—
Kankarn^19^	Y	Y	Y	Y	N	Y	N	Y	Y	Y	Y	Y	Y	Mental health support
Zhang^20^	Y	Y	Y	Y	Y	Y	N	Y	N	Y	Y	N	N	—
Fogazzi et al^21^—Togo	NR	NR	NR	Y	Y	Y	NR	NR	NR	NR	NR	NR	NR	—
Fogazzi et al^21^—Benin	NR	NR	NR	Y	NR	Y	NR	NR	NR	NR	NR	NR	NR	—
Njeri^22^	NR	NR	NR	NR	NR	NR	NR	NR	NR	NR	NR	NR	NR	—
Bello et al^23^	NR	NR	NR	NR	NR	NR	NR	NR	NR	NR	NR	NR	NR	—
Marie Patrice^24^	NR	NR	NR	NR	NR	NR	NR	NR	NR	NR	NR	NR	NR	

*Note.* CKD = chronic kidney disease; MBD = mineral and bone disorders; BP = blood pressure; DM = diabetes mellitus; CVD = cardiovascular disease; Y = yes (green); N = no (red); NR = not reported (grey).

The less commonly provided services were anemia management (n = 16), mineral and bone disorders (n = 15), vaccinations (primarily Hepatitis B) (n = 14), financial advice and support (n = 11), conservative kidney management (n = 11), and transplant evaluation (n = 11). In models where transplant workup was not included in care, patients were often referred to larger medical centres which had transplant workup capability. Temporary dialysis catheters were more commonly used for vascular access in settings where CKD patients typically first present with acute kidney injury requiring urgent dialysis (eg, Ghana,^9^ India^8^).

In terms of other management support, social workers (where available) provided financial advice for patients who had difficulty affording some or all of their CKD care. In care models that did not have social workers, financial advice typically came from physicians, who provided information on non-governmental organizations and other government subsidies if available. In 1 care model in India, physicians occasionally provided medications for free to patients on compassionate grounds.^8^

Conservative kidney management (management of patients with kidney failure without dialysis or kidney transplant) was not explicitly provided in many of the CKD care models (n = 11). Of the programs that indicated some form of conservative kidney management, 1 CKD model in Mexico provided this type of care to all patients who were not undergoing dialysis or transplant, either by choice or due to cost.^10^ In 1 CKD model described in Ghana, patients were referred to the family medicine directorate in the same hospital for conservative kidney management and palliative care.^9^ In Sri Lanka, patients were referred to government hospitals which had conservative kidney management capability.^7^ The remaining 8 programs provided conservative kidney management either directly through their own CKD program or through referrals to other units or facilities. The exact components of this service were difficult to determine, but the overall goal of all these programs was symptom management and improving quality of life (see Table 3).

## Discussion

Our systematic review identified 18 studies drawn from 16 different countries, including 4 studies that were included in the previous review by Stanifer et al.^3^ The findings provide comprehensive information on different models of care for CKD in LMICs. Most of the identified models of care in LMICs address fundamental aspects of CKD care, such as diabetes and hypertension management, prevention and treatment of metabolic bone disease, and anemia management, as recommended by KDIGO.^4^ More resource-intensive components such as workup for kidney transplantation or conservative kidney management were not offered at most of the programs in our review (Table 3). Instead, participants needed referral to specialized facilities to access these services. Although this is rational given limited access to kidney transplant services in many LMICs, this potential barrier may need to be addressed if expanded access to living donor transplantation is to become more common in LMICs as has been recommended.^25^

Nephrologists, other generalist or trainee physicians, nurses, and allied health professionals like dieticians and pharmacists were all commonly involved in the CKD programs we identified, whereas community health workers and social workers were included in a minority of programs (Table 2 and Figure 2). Having a multidisciplinary team, involving allied health professionals like dieticians and pharmacists is critical for effective CKD care. Dietary interventions like salt restriction and low protein diet are commonly used to mitigate CKD complications like volume overload and to slow the progression of CKD.^26^ Monitoring for medication interactions and dosing at low glomerular filtration rate (GFR) by pharmacists are also important to prevent further kidney injury in CKD.^27^ These interventions can slow CKD progression in many patients, delaying ESKD and the need for kidney replacement for as long as possible. Increasing capacity for social workers and community health workers to participate in CKD care will enhance the continuity of care and social supports for patients with kidney disease, by perhaps improving their access to beneficial treatments and services.

Although some elements of recommended CKD care were nominally available to patients, there likely were financial barriers that prevented full access for many patients. The different LMICs in our review used a range of funding models for CKD care, from full coverage of all services (eg, Thailand,^13^ Sri Lanka,^7^ and Malawi^14^) to being entirely paid for out-of-pocket by patients (eg, Benin,^21^ Togo,^21^ and Nigeria^23^). Consequently, a patient’s access to all components of CKD care varied by setting. In Ghana, for example, there is partial coverage of CKD care by the NHIS for hypertension and diabetes. However, there is no coverage for intravenous iron and erythropoietin stimulating agents for anemia care, temporary central lines, dialysis, and transplant. These are all services that patients will have to find funding for—either through private insurance (from their employer or purchased personally) or paid for entirely out-of-pocket.^9^ In countries like Nigeria,^23^ Togo,^21^ and Benin,^21^ no element of CKD care is paid for by the government. In Mexico, funding for CKD care takes several forms: about half of Mexico’s population has health care coverage through 3 main systems—Instituto Mexicano del Seguro Social (IMSS) provides health coverage for patients who work in the formal private sector. The Instituto de Seguridad y Servicios Sociales para los Trabajadores Estado (ISSSTE) provides health coverage to patients who are federal public workers, and patients who work for the Army receive health care coverage through their employer. Only patients who have health insurance through any of these systems have universal health coverage, including coverage for CKD and ESKD care. For the remaining half who do not have health insurance, there is a voluntary scheme through the Ministry of Health, called Seguro Popular which provides coverage for many conditions, but does not cover CKD or ESKD care.^28,29^

For patients who had to pay out-of-pocket for CKD care and did not have the resources to do so, financial support and counseling was provided by the physicians at many of the CKD clinics we studied; a minority were able to provide advice from a social worker (Tables 2 and 3). Social workers are a valuable addition to any CKD clinic, but may be especially helpful in low resource settings. Social workers can assist with patient advocacy, help patients and families with finding financial resources, and/or may provide support for managing the psychosocial stressors associated with chronic illness. Available evidence suggests that multidisciplinary CKD care (including input from social workers and other allied health professionals like community health workers and dieticians) helps to improve patient outcomes and satisfaction with care. Multidisciplinary care has been shown to be associated with a decrease in the decline in eGFR over time,^30^ up to 50% reduction in risk of death,^31^ and 40% reduction in risk of hospitalization secondary to infections compared with patients in non-multidisciplinary clinics.^32^

Finally, our study shows that there is a scarcity of data on how conservative kidney management is provided in LMICs, as many of the CKD care models did not highlight conservative kidney management as part of their model of care (Table 3). This may be because conservative kidney management is not as well established in LMICs, compared with traditional CKD care or dialysis. Nevertheless, there is strong evidence for the benefit of conservative kidney management, including improved symptom management^33^ and an improved overall quality of life.^34^ Given the high numbers of people in LMICs who do not have access to kidney replacement therapies, establishing and strengthening conservative kidney management should be a high priority.

In future, studies that highlight the importance of financially investing in CKD care in LMICs are needed, to reduce the undue burden of out-of-pocket expenses placed on patients, many of whom are already financially limited by other stressors posed by their illness, for example, loss of employment. Investment should also be geared toward establishing multidisciplinary CKD care teams, to improve mortality and morbidity outcomes for patients. In addition, research is needed to explore the feasibility of conservative kidney management in LMICs. As LMICs gradually work toward increasing investment in kidney care, the establishment of conservative kidney management in these countries will provide an opportunity to maintain an acceptable quality of life for patients who are currently either unable or unwilling to undergo dialysis or transplant.

Ultimately, no 1 CKD model of care will be appropriate for all settings in LMICs. Although our suggestions are based on available evidence for effective CKD care, each LMIC will have to adapt these recommendations to suit their unique circumstances and available resources as well as their broader health priorities. Input from local CKD clinics will be invaluable in this process, to individualize models of CKD care that are best suited to the patient population being served. In addition, it will likely be helpful to incorporate CKD care into national NCD strategies to reduce duplicated effort and create synergies. This will require collaboration between kidney health professionals, health professionals responsible for the prevention and control of other chronic illnesses, and regional/national health authorities.

Our review has several limitations that should be considered when interpreting results. First, many of the studies we included were not primarily focused on CKD care models in their respective countries. This meant that, we had to read through and extract information that referred to the model of care at the facility where the studies were conducted. If we were missing any information, we made reasonable effort to contact the authors of the studies (through email, Skype, Zoom, and telephone calls). Unfortunately, we were unable to reach the authors of 4 studies and so have limited information of the CKD model of care described in their study. Many of the papers did not have detailed information on the clinic models themselves, in terms of their setting, primary purpose, and patient population (eg, if they were early or advanced CKD clinics). The details we could glean from the papers and interviews have been included in Table 1. If key components of CKD care (eg, conservative kidney management) were not reported in a particular study, we were limited in our ability to comment on the overall impact on patients with CKD, as many of the studies did not discuss this in detail. Second, our systematic review included only 18 studies from 16 different LMICs. In addition, each study only described CKD care in the specific facility in which the study was conducted. Hence, our data cannot be seen as a representation of the reality of CKD care in LMICs in general, or in the specific countries in which the CKD care models were described. We tried to increase the yield of our search, by including studies from the Stanifer et al^3^ paper that met our inclusion criteria. By doing so, we acknowledge that we may have missed some studies which were not included in the original Stanifer et al^3^ review but may have been relevant to us. Third, given that our definition of a CKD care model was different from the definition used by Stanifer et al,^3^ we acknowledge that it may appear that there has been a shift in focus between both reviews. However, we feel that this is justified because our definition is more inclusive of care models that are multidisciplinary and are aimed at addressing important elements of CKD care as defined by KDIGO. Finally, we decided a priori to use KDIGO guidelines as our framework to evaluate CKD care models in LMICs. However, we recognize that there may be important elements of CKD care in LMICs that are not adequately addressed in the KDIGO guidelines, such as determining the etiologies of CKD in LMICs (where there are many other causes of CKD apart from diabetes and hypertension), evaluating the quality of CKD care, and establishing a CKD registry, among others. Similarly, it could also be argued that not all of the elements of CKD care as defined by KDIGO are equal in terms of their importance. Individual LMICs will have to determine, based on their individual circumstances, which aspects of CKD care they should emphasize in their care models.

## Conclusions

Our study adds to the body of literature on CKD in LMICs by showing that basic elements of CKD care are already delivered in a variety of different settings across multiple LMICs. However, access to certain specialized elements of care appears to depend in part on how CKD care is funded, either by the government or by the patients themselves. In addition, despite the obvious potential for benefit to patients, many CKD models do not include key allied health professionals like social workers and community health workers. Although it will undoubtedly be challenging for many LMICs to fund these additional health professionals, it seems likely that there will be good return-on-investment associated with such funding, especially given the high and growing need for conservative kidney management in LMICs. However, we acknowledge that the lack of high-quality data in this field would make future decisions on health care funding challenging for many LMICs. Our findings will be useful at the local level to CKD clinic managers and local health authorities, and at the national level to health policy officials who are responsible for prevention and control of CKD and other NCDs.

## Supplemental Material

sj-docx-1-cjk-10.1177_20543581221077505 – Supplemental material for Update on Existing Care Models for Chronic Kidney Disease in Low- and Middle-Income Countries: A Systematic Review Click here for additional data file. Supplemental material, sj-docx-1-cjk-10.1177_20543581221077505 for Update on Existing Care Models for Chronic Kidney Disease in Low- and Middle-Income Countries: A Systematic Review by Victoria Nkunu, Natasha Wiebe, Aminu Bello, Sandra Campbell, Elliot Tannor, Cherian Varghese, John Stanifer and Marcello Tonelli in Canadian Journal of Kidney Health and Disease

sj-docx-2-cjk-10.1177_20543581221077505 – Supplemental material for Update on Existing Care Models for Chronic Kidney Disease in Low- and Middle-Income Countries: A Systematic Review Click here for additional data file. Supplemental material, sj-docx-2-cjk-10.1177_20543581221077505 for Update on Existing Care Models for Chronic Kidney Disease in Low- and Middle-Income Countries: A Systematic Review by Victoria Nkunu, Natasha Wiebe, Aminu Bello, Sandra Campbell, Elliot Tannor, Cherian Varghese, John Stanifer and Marcello Tonelli in Canadian Journal of Kidney Health and Disease
