# Supplementary material for: Update on Existing Care Models for Chronic Kidney Disease in Low- and Middle-Income Countries: A Systematic Review
Source: Can J Kidney Health Dis. 2022 Mar 2;9:20543581221077505. doi: 10.1177/20543581221077505 (PMC8894943; doi:10.1177/20543581221077505)
Supplement: sj-docx-1-cjk-10.1177_20543581221077505 – Supplemental material for Update on Existing Care Models for Chronic Kidney Disease in Low- and Middle-Income Countries: A Systematic Review [file sj-docx-1-cjk-10.1177_20543581221077505.docx]

**APPENDIX**

**Supplemental Table S1. Search strategies**

| Database and Platform | Search Strategies | Date and Coverage |
| --- | --- | --- |
| MEDLINE (OVID) | 1. exp Community Health Services/ or exp Health Services, Indigenous/ or exp Primary Health Care/ or exp Rural Health Services/ or exp Telemedicine/ or telemedicine.ti,ab. or ((exp Disease Management/ or exp Health Promotion/ or exp Nutrition Therapy/ or exp Community Health Workers/ or management.ti,ab. or health promotion.ti,ab. or education.ti,ab. or multidisciplinary.ti,ab. or integrated.ti,ab. or transdisciplinary.ti,ab. or participatory.ti,ab. or community.ti,ab. or rural.ti,ab. or outreach.ti,ab. or ambulatory.ti,ab. or nurse.ti,ab. or nursing.ti,ab. or pharmacist.ti,ab. or pharmacists.ti,ab. or traditional.ti,ab. or healer.ti,ab. or community.ti,ab. or general practice.ti,ab. or tertiary.ti,ab. or primary.ti,ab. or outpatient.ti,ab.) and (exp Delivery of Health Care/ or exp Ambulatory Care/ or clinic.ti,ab. or service.ti,ab. or services.ti,ab. or model.ti,ab. or models.ti,ab. or program.ti,ab. or programs.ti,ab. or intervention.ti,ab.))  2. exp Renal Insufficiency, Chronic/ or chronic kidney.ti,ab. or chronic renal.ti,ab. or CKD.ti,ab. or CRD.ti,ab. or exp proteinuria/ or proteinuria.ti,ab. or albuminuria.ti,ab.  3. egypt/ or morocco/ or tunisia/ or cameroon/ or central african republic/ or chad/ or congo/ or "democratic republic of the congo"/ or equatorial guinea/ or gabon/ or "sao tome and principe"/ or burundi/ or djibouti/ or eritrea/ or ethiopia/ or kenya/ or rwanda/ or somalia/ or south sudan/ or sudan/ or tanzania/ or uganda/ or angola/ or lesotho/ or malawi/ or mozambique/ or swaziland/ or zambia/ or zimbabwe/ or benin/ or burkina faso/ or cabo verde/ or cote d'ivoire/ or gambia/ or ghana/ or guinea/ or guinea-bissau/ or liberia/ or mali/ or mauritania/ or niger/ or nigeria/ or senegal/ or sierra leone/ or togo/ or honduras/ or nicaragua/ or bolivia/ or kazakhstan/ or kyrgyzstan/ or tajikistan/ or uzbekistan/ or cambodia/ or laos/ or myanmar/ or philippines/ or timor-leste/ or vietnam/ or bangladesh/ or bhutan/ or india/ or afghanistan/ or syria/ or yemen/ or nepal/ or pakistan/ or sri lanka/ or "democratic people's republic of korea"/ or mongolia/ or borneo/ or melanesia/ or papua new guinea/ or vanuatu/ or haiti/ or comoros/ or madagascar/ or sri lanka/ or (Afghanistan or Afghani or Afghan or Angola* or Bangladesh* or Benin or Beninese or Bhutan or Bolivia* or Burkina Faso or Burkinabe or Burundi* or Cabo Verde or Cape Verde or Cambodia* or Cameroon* or Central African Republic or Chad or Chadian or Tchad or Comoros or Comoran or Congo or Congolese or Cote d'ivoire or Ivorian or Djibouti or Egypt or Egyptian or El Salvador or Salvadoran or Eritrea* or Ethiopia* or Gambia or Gambian or (Georgia not United States) or Ghana* or Guinea or Guinea Bissau* or Haiti or Haitian or Hondura* or India or (Indian not American) or Indonesia* or Kenya* or Kiribati or North Korea* or DPRK or Kosovo or Kosovar or Kosovan or Kyrgyz* or Laos or Laotian or Lesotho or Mosotho or Basotho or Liberia* or Madagascar or Malagasy or Malawi* or Mali or Malian or Mauritania* or Micronesia* or Moldova* or Mongolia* or Morocco or Moroccan or Mozambique or Mozambican or Myanmar or Burmese or Myanmarese or Nepal or Nepalese or Nicaragua* or Niger or Nigerien or Nigeria or Pakistan* or Papua New Guinea* or Philippines or Filipino* or Rwanda* or "Sao Tome and Principe" or San Tomean or Senegal* or Sierra Leone* or Solomon Island* or Somalia* or Sri Lanka* or Sudan or Sudanese or Swaziland or Swazi or Syria or Syrian or Tajikistan or Tajik or Tadzhik or Tanzania* or Timor Leste or Timorese or Togo or Togolese or Tunisia* or Uganda* or Ukraine or Ukrainian or Uzbekistan* or Uzbeki or Vanuatu or Vietnam* or West Bank or Gaza or Yemen* or Zambia* or Zimbabwe*).ti,ab,cp.  4. 1 and 2 and 3  5. limit 4 to (case reports or comment or editorial or letter)  6. 4 not 5  7. animal/ not (animal/ and human/)  8. 6 not 7  9. limit 8 to yr="2017 -Current" | Ovid MEDLINE® Epub ahead of print, In-process & other non-indexed citations, Ovid MEDLINE® Daily, and Ovid MEDLINE® 1946 to September 11, 2020  September 14, 2020 |
| EMBASE (OVID) | 1. exp community care/ or exp transcultural care/ or exp primary health care/ or exp rural health care/ or exp telemedicine/ or telemedicine.ab,ti. or ((exp disease management/ or exp health education/ or exp diet therapy/ or exp nutrition/ or exp health auxiliary/ or management.ab,ti. or health promotion.ab,ti. or education.ab,ti. or multidisciplinary.ab,ti. or integrated.ab,ti. or transdisciplinary.ab,ti. or participatory.ab,ti. or community.ab,ti. or rural.ab,ti. or outreach.ab,ti. or ambulatory.ab,ti. or nurse.ab,ti. or nursing.ab,ti. or pharmacist.ab,ti. or pharmacists.ab,ti. or traditional.ab,ti. or healer.ab,ti. or community.ab,ti. or general practice.ab,ti. or tertiary.ab,ti. or primary.ab,ti. or outpatient.ab,ti.) and (exp health care delivery/ or clinic.ab,ti. or service.ab,ti. or services.ab,ti. or model.ab,ti. or models.ab,ti. or program.ab,ti. or programs.ab,ti. or intervention.ab,ti.))  2. exp chronic kidney failure/ or exp proteinuria/ or chronic kidney.ab,ti. or chronic renal.ab,ti. or CKD.ab,ti. or CRD.ab,ti. or proteinuria.ab,ti. or albuminuria.ab,ti.  3. angola/ or benin/ or burkina faso/ or burundi/ or cameroon/ or cape verde/ or central african republic/ or chad/ or comoros/ or congo/ or cote d'ivoire/ or democratic republic congo/ or djibouti/ or equatorial guinea/ or eritrea/ or ethiopia/ or gabon/ or gambia/ or ghana/ or guinea/ or guinea-bissau/ or kenya/ or lesotho/ or liberia/ or madagascar/ or malawi/ or mali/ or mozambique/ or namibia/ or niger/ or nigeria/ or rwanda/ or senegal/ or sierra leone/ or exp somalia/ or south sudan/ or sudan/ or swaziland/ or tanzania/ or togo/ or uganda/ or zimbabwe/ or algeria/ or egypt/ or mauritania/ or morocco/ or tunisia/ or el salvador/ or honduras/ or nicaragua/ or bolivia/ or syrian arab republic/ or yemen/ or haiti/ or exp "georgia (republic)"/ or kosovo/ or moldova/ or Mongolia/ or "sao tome and principe"/ or exp ukraine/ or afghanistan/ or bangladesh/ or bhutan/ or exp india/ or nepal/ or exp pakistan/ or sri lanka/ or kyrgyzstan/ or tajikistan/ or uzbekistan/ or cambodia/ or laos/ or myanmar/ or papua new guinea/ or timor-leste/ or viet nam/ or "federated states of micronesia"/ or kiribati/ or philippines/ or solomon islands/ or vanuatu/ or North Korea/ or "sao tome and principe"/ or (Afghanistan or Afghani or Afghan or Angola* or Bangladesh* or Benin or Beninese or Bhutan or Bolivia* or Burkina Faso or Burkinabe or Burundi* or Cabo Verde or Cape Verde or Cambodia* or Cameroon* or Central African Republic or Chad or Chadian or Tchad or Comoros or Comoran or Congo or Congolese or Cote d'ivoire or Ivorian or Djibouti or Egypt or Egyptian or El Salvador or Salvadoran or Eritrea* or Ethiopia* or Gambia or Gambian or (Georgia not United States) or Ghana* or Guinea or Guinea Bissau* or Haiti or Haitian or Hondura* or India or (Indian not American) or Indonesia* or Kenya* or Kiribati or North Korea* or DPRK or Kosovo or Kosovar or Kosovan or Kyrgyz* or Laos or Laotian or Lesotho or Mosotho or Basotho or Liberia* or Madagascar or Malagasy or Malawi* or Mali or Malian or Mauritania* or Micronesia* or Moldova* or Mongolia* or Morocco or Moroccan or Mozambique or Mozambican or Myanmar or Burmese or Myanmarese or Nepal or Nepalese or Nicaragua* or Niger or Nigerien or Nigeria or Pakistan* or Papua New Guinea* or Philippines or Filipino* or Rwanda* or "Sao Tome and Principe" or San Tomean or Senegal* or Sierra Leone* or Solomon Island* or Somalia* or Sri Lanka* or Sudan or Sudanese or Swaziland or Swazi or Syria or Syrian or Tajikistan or Tajik or Tadzhik or Tanzania* or Timor Leste or Timorese or Togo or Togolese or Tunisia* or Uganda* or Ukraine or Ukrainian or Uzbekistan* or Uzbeki or Vanuatu or Vietnam* or West Bank or Gaza or Yemen* or Zambia* or Zimbabwe*).ti,ab,cp.  4. 1 and 2 and 3  5. limit 4 to (editorial or letter or note)  6. 4 not 5  7. animal/ not (animal/ and human/)  8. 6 not 7  9. limit 8 to yr="2017 -Current" | Embase 1974 to September 11, 2020  September 14, 2020 |
| Global Health  (OVID) | 1. exp Community Health Services/ or exp Health Services, Indigenous/ or exp Primary Health Care/ or exp Rural Health Services/ or exp Telemedicine/ or telemedicine.ti,ab. or ((exp Disease Management/ or exp Health Promotion/ or exp Nutrition Therapy/ or exp Community Health Workers/ or management.ti,ab. or health promotion.ti,ab. or education.ti,ab. or multidisciplinary.ti,ab. or integrated.ti,ab. or transdisciplinary.ti,ab. or participatory.ti,ab. or community.ti,ab. or rural.ti,ab. or outreach.ti,ab. or ambulatory.ti,ab. or nurse.ti,ab. or nursing.ti,ab. or pharmacist.ti,ab. or pharmacists.ti,ab. or traditional.ti,ab. or healer.ti,ab. or community.ti,ab. or general practice.ti,ab. or tertiary.ti,ab. or primary.ti,ab. or outpatient.ti,ab.) and (exp Delivery of Health Care/ or exp Ambulatory Care/ or clinic.ti,ab. or service.ti,ab. or services.ti,ab. or model.ti,ab. or models.ti,ab. or program.ti,ab. or programs.ti,ab. or intervention.ti,ab.))  2. exp Renal Insufficiency, Chronic/ or chronic kidney.ti,ab. or chronic renal.ti,ab. or CKD.ti,ab. or CRD.ti,ab. or exp proteinuria/ or proteinuria.ti,ab. or albuminuria.ti,ab.  3. egypt/ or morocco/ or tunisia/ or cameroon/ or central african republic/ or chad/ or congo/ or "democratic republic of the congo"/ or equatorial guinea/ or gabon/ or "sao tome and principe"/ or burundi/ or djibouti/ or eritrea/ or ethiopia/ or kenya/ or rwanda/ or somalia/ or south sudan/ or sudan/ or tanzania/ or uganda/ or angola/ or lesotho/ or malawi/ or mozambique/ or swaziland/ or zambia/ or zimbabwe/ or benin/ or burkina faso/ or cabo verde/ or cote d'ivoire/ or gambia/ or ghana/ or guinea/ or guinea-bissau/ or liberia/ or mali/ or mauritania/ or niger/ or nigeria/ or senegal/ or sierra leone/ or togo/ or honduras/ or nicaragua/ or bolivia/ or kazakhstan/ or kyrgyzstan/ or tajikistan/ or uzbekistan/ or cambodia/ or laos/ or myanmar/ or philippines/ or timor-leste/ or vietnam/ or bangladesh/ or bhutan/ or india/ or afghanistan/ or syria/ or yemen/ or nepal/ or pakistan/ or sri lanka/ or "democratic people's republic of korea"/ or mongolia/ or borneo/ or melanesia/ or papua new guinea/ or vanuatu/ or haiti/ or comoros/ or madagascar/ or sri lanka/ or (Afghanistan or Afghani or Afghan or Angola* or Bangladesh* or Benin or Beninese or Bhutan or Bolivia* or Burkina Faso or Burkinabe or Burundi* or Cabo Verde or Cape Verde or Cambodia* or Cameroon* or Central African Republic or Chad or Chadian or Tchad or Comoros or Comoran or Congo or Congolese or Cote d'ivoire or Ivorian or Djibouti or Egypt or Egyptian or El Salvador or Salvadoran or Eritrea* or Ethiopia* or Gambia or Gambian or (Georgia not United States) or Ghana* or Guinea or Guinea Bissau* or Haiti or Haitian or Hondura* or India or (Indian not American) or Indonesia* or Kenya* or Kiribati or North Korea* or DPRK or Kosovo or Kosovar or Kosovan or Kyrgyz* or Laos or Laotian or Lesotho or Mosotho or Basotho or Liberia* or Madagascar or Malagasy or Malawi* or Mali or Malian or Mauritania* or Micronesia* or Moldova* or Mongolia* or Morocco or Moroccan or Mozambique or Mozambican or Myanmar or Burmese or Myanmarese or Nepal or Nepalese or Nicaragua* or Niger or Nigerien or Nigeria or Pakistan* or Papua New Guinea* or Philippines or Filipino* or Rwanda* or "Sao Tome and Principe" or San Tomean or Senegal* or Sierra Leone* or Solomon Island* or Somalia* or Sri Lanka* or Sudan or Sudanese or Swaziland or Swazi or Syria or Syrian or Tajikistan or Tajik or Tadzhik or Tanzania* or Timor Leste or Timorese or Togo or Togolese or Tunisia* or Uganda* or Ukraine or Ukrainian or Uzbekistan* or Uzbeki or Vanuatu or Vietnam* or West Bank or Gaza or Yemen* or Zambia* or Zimbabwe*).ti,ab,cp.  4. 1 and 2 and 3  5. limit 4 to yr="2017 -Current" | Global Health 1910 to 2020 Week 36  September 14, 2020 |
