# Supplementary material for: Update on Existing Care Models for Chronic Kidney Disease in Low- and Middle-Income Countries: A Systematic Review
Source: Can J Kidney Health Dis. 2022 Mar 2;9:20543581221077505. doi: 10.1177/20543581221077505 (PMC8894943; doi:10.1177/20543581221077505)
Supplement: sj-docx-2-cjk-10.1177_20543581221077505 – Supplemental material for Update on Existing Care Models for Chronic Kidney Disease in Low- and Middle-Income Countries: A Systematic Review [file sj-docx-2-cjk-10.1177_20543581221077505.docx]

Please assess each screened-in article according to the criteria below.

**Preliminary:**

1a. Is the report written in English or French? **Yes** **No**

1b. Is this a full manuscript? **Yes** **No**

[ ] peer-reviewed and published, or

[ ] grey literature from a non-private source

1d. Was the population of interest human? **Yes** **No**

**Population:**

2a. was the population of interest largely adults? **Yes** **No**

2b. Was the population/subgroup of interest on chronic dialysis **Yes** **No**

or end-stage?

[ ] hemodialysis, or

[ ] peritoneal dialysis

2c. Was the population of interest residing in a LMIC **Yes** **No**

(see World Bank list)?

**Care Model (one or more of A or B or C are sufficient for inclusion):**

3a. Does the manuscript describe how dialysis is organized, **Yes** **No**

funded, staffed, financed, delivered or regulated?

OR

3b. Was there a description of how the technical aspects of

dialysis are provided (e.g., dialysis water treatment, who is **Yes** **No**

actually doing the dialysis exchanges for PD)?

OR

3c. Was there a description of how patients are selected to receive **Yes** **No**

dialysis, transported to dialysis, supported in receiving dialysis

(e.g., by family members or paid volunteers), or to choose when to initiate

dialysis?

**Final decision**:

Should this study be included in the next stage? **Yes** **No**

(Answer yes if all the above are yes)

# Unsure

*Put into Unsure group for consensus*

**Consensus decision:**

**Yes** **No 3^rd^ Party**

If this is a systematic review or abstract, could the included studies or full manuscript be relevant?
